# Assessing the impact of mHealth interventions in low- and middle-income countries – what has been shown to work?

**DOI:** 10.3402/gha.v7.25606

**Published:** 2014-10-27

**Authors:** Charles S. Hall, Edward Fottrell, Sophia Wilkinson, Peter Byass

**Affiliations:** 1 UCL Medical School, London, UK; 2 UCL Institute for Global Health, London, UK; 3 Department of Public Health and Clinical Medicine, Umeå Centre for Global Health Research, Umeå University, Umeå, Sweden; 4 BBC Media Action, London, UK; 5 Medical Research Council/Wits University Rural Public Health and Health Transitions Research Unit (Agincourt), School of Public Health, Faculty of Health Sciences, University of the Witwatersrand, Johannesburg, South Africa

**Keywords:** mHealth, low- and middle-income countries, health systems, interventions, mobile data

## Abstract

**Background:**

Low-cost mobile devices, such as mobile phones, tablets, and personal digital assistants, which can access voice and data services, have revolutionised access to information and communication technology worldwide. These devices have a major impact on many aspects of people's lives, from business and education to health. This paper reviews the current evidence on the specific impacts of mobile technologies on tangible health outcomes (mHealth) in low- and middle-income countries (LMICs), from the perspectives of various stakeholders.

**Design:**

Comprehensive literature searches were undertaken using key medical subject heading search terms on PubMed, Google Scholar, and grey literature sources. Analysis of 676 publications retrieved from the search was undertaken based on key inclusion criteria, resulting in a set of 76 papers for detailed review. The impacts of mHealth interventions reported in these papers were categorised into common mHealth applications.

**Results:**

There is a growing evidence base for the efficacy of mHealth interventions in LMICs, particularly in improving treatment adherence, appointment compliance, data gathering, and developing support networks for health workers. However, the quantity and quality of the evidence is still limited in many respects.

**Conclusions:**

Over all application areas, there remains a need to take small pilot studies to full scale, enabling more rigorous experimental and quasi-experimental studies to be undertaken in order to strengthen the evidence base.

Jeffrey Sachs, the Director of the Earth Institute, has suggested that ‘Mobile phones and wireless internet end isolation, and will therefore prove to be the most transformative technology of economic development of our time’ (1). The mHealth community believes that this extends to healthcare. This review aims to summarise and assess the evidence of impacts that mobile technologies have had on improving health in countries categorised by the World Bank as low- and middle-income (LMICs) (2), through mHealth (mobile health) interventions. The World Health Organization (WHO) has defined mHealth as ‘Medical and public health practice supported by mobile devices, such as mobile phones, patient monitoring devices, personal digital assistants, and other wireless devices’ (3).

In many places people are more likely to have access to a mobile phone than to have clean water, a bank account, or even a source of electricity (4). Although, in richer countries, there has been a shift from landline-based technologies towards mobile telecommunications, many LMICs have made a technological leap with a ‘mobile-first’-based approach to communications. The market penetration of mobile phones has been estimated to reach 78% worldwide (5).

Although low-cost mobile devices provide new and potentially transformative opportunities for all those working to improve health outcomes, nevertheless, to date, there is little evidence on mHealth interventions that have been implemented in LMICs and assessed in terms of specific outcomes. Mobile network coverage and availability of mobile handsets are necessary but not sufficient conditions to capitalise on possible mHealth benefits (6). Despite industry optimism, mobile-first strategies tend to bypass some of the traditional features of landline-based systems, such as effective emergency call systems for health crises. An extensive review and meta-analysis of mHealth interventions aimed at improving healthcare delivery identified 42 controlled trials, but concluded that none delivered high-quality evidence and almost all were in high-income countries (7). Hence this review focuses specifically on LMICs.

Mobile phones and mobile technologies have moved beyond calls, simple short messaging service (SMS) text and voice messaging, to incorporate mobile Internet browsing, Voice over Internet Protocol services (e.g. Skype), instant messaging services, photographic capabilities, and a wide variety of device-based software applications (commonly known as ‘apps’). These various mobile technology functionalities offer a range of opportunities for mHealth interventions, summarised in Box 1, from health promotion via SMS texts and interactive voice response campaigns and content to mobile phone-based imaging (which has potential diagnostic capabilities).

Box 1 **Common application domains for mHealth (8)**

1. Client Education and Behaviour Change
2. Sensors and Point of Care Diagnostics
3. Registries and Vital Events Tracking
4. Data Collection and Reporting
5. Electronic Health Records
6. Electronic Decision Support: Information, Protocols, Algorithms, Checklists
7. Provider-Provider Communication: User Groups, Consultation
8. Provider Work Planning and Scheduling
9. Provider Training and Education
10. Human Resource Management
11. Supply Chain Management
12. Financial Transactions and Incentives

This paper is a review of evidence of the health impacts of mHealth interventions, categorised by the 12 common applications of mHealth as described by Labrique et al. (8) (Box 1) in LMICs. We chose this framework as one of the few pieces of established work in this field, in preference to reinventing it. For the purposes of this review, health impacts were defined *a priori* in terms of measurable changes in mortality, morbidity, disability adjusted life years (DALYs), and improved disease detection rates. We also included behaviour change as a valid health impact in this review where changes in knowledge, self-efficacy, attitudes, or behaviours themselves had a reasonably direct association with improved health, such as improved antenatal care uptake, or reduced health personnel absenteeism. Given that previous work found little high-quality evidence of mHealth outcomes, even in high-income settings, we chose to take an inclusive view of sources presenting effects of mHealth applications in LMICs, rather than insisting on particular levels of evidence.

## Methods

**Fig. 1:**
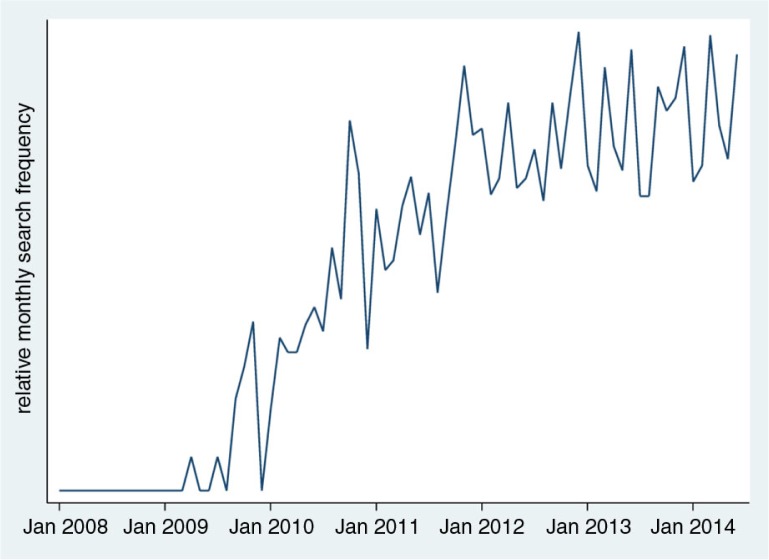
Google Trends searches for ‘mHealth’ over time (data sourced from Google Trends 25 July 2014).

An initial search of Google Trends (which monitors the historical use of Internet search terms) for the term ‘mHealth’ revealed time trends in searches over the period during which mHealth has come into parlance. Figure 1 shows that Google searches for mHealth were first used in 2009, and plateaued at current levels in 2013. Based on these results, papers published before 2009 were excluded from this review.

A systematic electronic literature search from 2009 to early 2014 was undertaken using the online databases PubMed and Google Scholar. The search term ‘Health’ was included with a combination of the following medical subject headings: one or more of (‘mHealth’, ‘mobile phone*’, ‘cellphone*’, ‘cellular phone’, ‘mobile health’, and ‘mobile telemedicine’); and one or more of (‘developing’, ‘resource-limited’, ‘low-income country*’, ‘middle-income country*’, ‘impact’, and ‘evidence’). Because of the paucity of published literature on mHealth outcomes in LMICs, the search was extended to include grey literature (according to the Luxembourg Definition ‘that which is produced on all levels of government, academics, business, and industry in print and electronic formats, but which is not controlled by commercial publishers’) found in the key mHealth online databases mHealth Evidence (9), GSMA (10), and the Global mHealth Initiative (11).

**Fig. 2:**
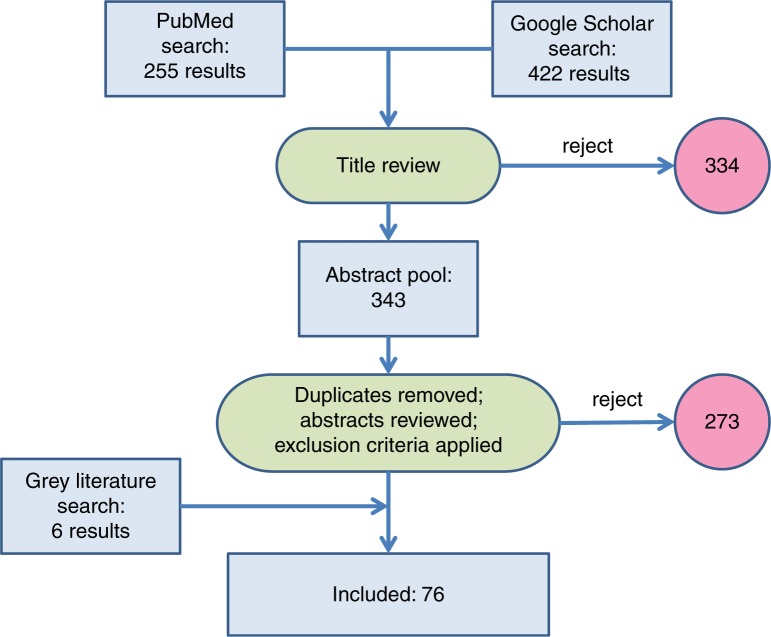
Search and exclusion process for literature review.

Duplicate results were removed using Endnote software. One author (CSH) also performed a manual search to remove duplicates. The searches resulted in 676 papers found on PubMed and Google Scholar, and another six relevant, non-duplicated papers discovered from grey literature sources (Fig. 2). For these 676 results, abstracts were screened for relevance and selected for full text review. The exclusion criteria included projects not relating to human health, projects that did not use technology as per the WHO definition above, studies from high-income countries, studies published before 2009, and studies that did not have evidence of a measured change in health outcomes (defined as mortality, morbidity, DALYs, and behavioural change). In total, 76 papers were included in the review after this process and were cross-checked by another author (PB).

## Results

The 76 reviewed papers are categorised in Table 1 according to type of publication and geographical origin. Findings from the 76 reviewed papers are then presented under the application domains identified in Box 1. There were no results uniquely associated with two of those domains (human resource management, and financial transactions and incentives) and the very limited material relating to registries and vital events tracking has been subsumed under data collection and reporting.

**Table 1:** Types of paper and geographical origin for 76 mHealth publications identified from the literature search

	Peer-reviewed research articles	Review articles	Internet and grey literature sources
Africa	34 publications: Benin (12); Botswana (13–16); Egypt (17); Kenya (18–24); Malawi (25); Nigeria (26); Rwanda (27–29); South Africa (30–33); Swaziland (34, 35); Tanzania (36–38); Uganda (39–45)		5 publications: Malawi (46); Rwanda (47); Uganda (48–50)
Americas	5 publications: Brazil (51); Mexico (52); Peru (53–55)		
Asia	9 publications: Cambodia (56); China (57); Indonesia (58, 59); India (60, 61); Pakistan (62); Thailand (63, 64)		2 publications: Bangladesh (65); India (66)
Europe			1 publication: Kosovo (67)
Non-geographic	7 publications: (8, 68–73)	6 publications: (74–79)	7 publications: (3–5, 80–83)

### Client education and behaviour change

This was the most represented domain, to which 20 of the reviewed papers related. As a consequence of high uptake of mobile phones in LMICs, mobile-based educational schemes have an opportunity to enhance health behaviour, and therefore health outcomes, at the population level. Educational services and campaigns might aim to increase target groups’ knowledge and shift behaviour and norms around different health issues, services, and products. The area of treatment adherence in particular has received much attention. Mobile technologies make people more contactable and as such offer a useful tool to deliver education and improve health-seeking behaviour or health-related lifestyle decisions.

A risk-benefit analysis study in Thailand exploring inexpensive mHealth reminders to improve TB medication adherence showed increased mortality and DALYs compared to equally inexpensive family-member-based directly observed treatments (63). Retrospective analysis of an intervention in South Africa using SMS reminders sent to 18 tuberculosis patients who delayed in opening their wireless pill bottles showed improved health compared to 72 control patients who received no SMS reminder (31). The study suggests the utility of mHealth strategies for increasing effectiveness and thereby decreasing overall costs to the health system. A systematic review of one randomised controlled trial (RCT) in Argentina and three non-randomised controlled trials in South Africa and Kenya suggested there was low-quality evidence (due to high risk of bias and study heterogeneity) and hence inconclusive evidence on improved TB treatment adherence via mHealth interventions (78). The authors suggested the need for more rigorous studies, especially randomised controlled trials, before conclusions could be drawn on the efficacy of mHealth interventions to improve TB medication adherence.

Systematic analysis of RCTs on SMS interventions to improve HIV anti-retroviral treatment (ART) adherence showed that mHealth interventions helped to reduce viral load amongst HIV sufferers by improving adherence (20, 71, 76). Studies showed the importance of the frequency and timing of messages to users to optimize their efficacy (20, 71). However, an RCT in China discussed how voice calls showed no statistically significant improvement in ART adherence (57).

mHealth education and behaviour change initiatives have also targeted non-communicable diseases in LMICs, although evidence of effectiveness is both mixed and scant. A before-and-after evaluation of a mHealth-based peer-support group for women suffering from diabetes mellitus in South Africa was undertaken. Women in the study were linked with a ‘buddy’ who provided support by exchanging SMS text messages. Health change was measured by body mass index reduction, improved health-seeking behaviours, blood glucose levels, and increases in coping. Results showed no evidence of significant change in health over a 6-month period, although the sample size was very small (*n*=22) (32). Meanwhile, an RCT in southern India concluded that using SMS could reduce the incidence of type 2 diabetes in Indian Asian men with impaired glucose tolerance. In this trial, one group at risk of type 2 diabetes was randomly assigned to receive numerous text messages with educational and motivational advice to help them to adopt a healthier lifestyle over a 3-year period, whereas the control group received only standard lifestyle modification advice at the start of the trial. By trial end, 18% of participants in the intervention group had developed type 2 diabetes compared with 27% in the control group (61).

A systematic review of the literature identified that although SMS campaigns have been useful in improving treatment compliance, they failed to address the risky health behaviour factors associated with contracting HIV, and did not improve uptake of HIV treatment (79). Studies offering free HIV tests for correct answers in an SMS quiz on HIV in Uganda demonstrated two major issues. First, poor results on uptake were noted, with only 2.3% of recipients opting to redeem their free HIV test (41). Second, equity issues were raised. Vulnerable groups were less likely to know correct answers to the quiz, therefore less likely to benefit from the scheme (40). In this context, women were less likely to own a mobile phone, so were less likely to directly benefit from SMS education (40, 41, 77). Furthermore it has been suggested (but not proven) that SMS sexual education increased self-reported risky sexual behaviour amongst intervention groups (48). It was not clear, however, if this was an actual change in behaviour, or a change in reporting because of improved understanding of what constituted risky behaviour.

Increasing knowledge and shifting behaviour around family planning and contraception is an important health issue. An SMS contraceptive education scheme in Kenya suggested improved contraceptive knowledge and use, but suffered from a sample size that was too small, with no control group, thus reducing the ability to draw firm conclusions (22). A Peruvian RCT, using educational SMS to reduce risks of contracting dengue fever, hinted at improved educational equity via SMS but showed no statistically significant improvement over alternative educational schemes (54).

Antenatal care uptake is an important consideration in maternal and child health (74). A cluster-randomised controlled trial in Zanzibar, Tanzania, of 2,550 women (intervention arm 1,311 vs. control 1,239) investigated the effect of improving communications between midwives and pregnant women via mobile phones. Patients were given contact details of a midwife and prepaid credit to pay for communications. Results showed that 60% of the intervention arm used a skilled birth attendant at the birth, compared to 47% in the control group (37). Antenatal care improved with 44% of the intervention arm receiving the recommended four antenatal visits compared to 31% in the control group (38). This demonstrated that mHealth can help to improve pregnancy management outcomes, as part of a wider antenatal system.

A scheme trialled in Kenya showed that conditional cash transfers, via mobile phone, and SMS reminders, improved child vaccination rates in rural areas (23). A significant proportion of mothers, however, were not allowed to partake in the scheme by their husbands, thus identifying a major potential barrier to mHealth interventions that would need to be taken into account during an intervention's design.

### Sensors and point of care diagnostics

Eight studies reported technical implementations related to imaging. There are some novel ideas around using the wealth of sensors available on, or as attachments for, mobile devices, aimed at improving medical diagnostics in resource-limited settings. Using digital communications to transmit images for remote expert interpretation could ameliorate shortages of specialist physicians in LMICs, especially in rural areas.

Mobile-based light microscopy offers a cheaper and more transportable tool for diagnosing infectious and haematological conditions (70, 73). Importantly, images can be captured by community health workers (CHWs) and transmitted wirelessly for diagnosis by doctors (45, 70). Results showed that phone cameras have high enough resolution to see key infectious agents and haematological signs. A feasibility study in Uganda demonstrated that data transfer via MMS and mobile Internet direct from the phone to a central database for reporting by trained pathologists was possible (45). More studies into improved diagnostic systems compared to standard methods in LMICs are still necessary, however, to demonstrate possible benefits.

The mobile phone camera has also been shown to be useful for dermatological diagnosis. Feasibility and applicability studies in Egypt and Uganda have demonstrated the possibilities of mobile tele-dermatology to improve diagnostic rates of dermatological conditions (17, 42). Larger scale studies are still needed, because of the small sample size in both studies (*n*=30 in Egypt and *n*=72 in Uganda).

In Botswana, women suspected to have cervical cancer had their cervices stained by trained health workers, then imaged by mobile phone and sent to be reported by trained gynaecologists (16). The results showed that the test and photography combined could only offer 70–81% concordance with visual inspection. The authors argued that the overall benefits from reduced referral delay and decreased travel times for patients would lead to overall improved health compared to the currently available options, but no evidence was offered to support this.

A scheme in Rwanda aimed to combine blood testing machines based on mobile phone technology with cloud-based medical records (27). The mobile blood testing devices cost US$1,000 compared to US$19,000 for bench-top machines. Results from 167 samples, comparing the new technology to gold standards, were encouraging with HIV, viral hepatitis, and sexually transmitted infection test sensitivities and specificities of 100 and 99%, respectively. Results were directly transmitted to a central computer server both via satellite and SMS, with 33/40 and 38/40 being successfully received by each method respectively. Cheaper and faster diagnostic tools combined with centralised health records offer a novel modality to improve health. However larger implementations and more rigorous user testing are needed, especially to establish the efficacy of mass data transmission of patient records.

### Data collection and reporting (including registries and vital events tracking)

Data collection and registration was the second most reported domain, with 17 reviewed papers. This domain pre-dates the mHealth concept, though here the same 2009–14 period has been reviewed. CHWs, equipped with mobile data collection tools, have been shown to collect higher quality data compared to using traditional paper resources in some settings. mHealth data collection has been shown to be more cost effective than paper resources (21), and lower rates of data loss were reported (33), with fewer errors (46, 52, 53). An exception was a Kenyan study which showed data accuracy to be ‘sub-optimal’, with only 58% of data reporting as falling within ±10% of a predetermined gold standard (19). mHealth tools can speed up the collection of data. UNICEF reported their RapidSMS tool, for monitoring malnutrition rates, eliminated delays of several months associated with transporting paper-based data (46).

In Senegal, data were collected from health posts on personal digital devices loaded with an 82-question survey based on the EpiSurveyor (now called Magpi) software (3). Collected data were sent to district and national offices for analysis. Findings of the surveys showed that 45% of surveyed districts were not using partographs to monitor labour, despite being an important tool proven to help reduce intrapartum birth complications. Policy was subsequently altered to improve partograph uptake, with additional EpiSurveyor results demonstrating 28% use of partographs in the pilot regions, compared to 1% in regions outside the EpiSurveyor pilot.

Cambodia, in conjunction with the WHO, has reported a programme set up in the wake of the SARS crisis of 2003, called ‘Cam e-Warn’, to improve health surveillance. Approximately 1,200 staff across the country are involved in collecting and analysing data on 12 monitored diseases (3). Data are analysed and transferred via SMS at local, district, provincial, and national office levels. When a threshold level of cases of any of the monitored diseases is reached, it leads to a warning being generated and rapid response teams are dispatched to the region to take action. Since its implementation in 2008 it has detected multiple outbreaks, including one of acute watery diarrhoea, a major cause of child mortality. Efforts are now being made to make the system more streamlined, to allow primary reporters to send SMS data directly to national offices, in an effort to improve efficiency. The low data capacity associated with using SMS has been highlighted as a barrier to its use for long health surveys (46).

There is overlap between mHealth data collection initiatives and the delivery of other health services such as immunisation programmes. SMS-based registration of birth dates and due dates in Bangladesh (65), Uganda, Nigeria, and Senegal (67) has been used to improve data on birth rates, whilst improving the coverage of timely child immunisation. This suggests positive effects of mHealth interventions, but the data on which these statements were made were not accessible, nor was there discussion of the study design.

Fast reporting of test results can enable faster treatment of medical issues and, in the case of communicable diseases, might prevent further infection. Logistic issues are a major barrier to fast test reporting; with laboratories frequently being far away from the clinics they serve. A survey in Uganda found the notification of abnormal blood test results via SMS to be an acceptable method amongst HIV-positive patients (44).

A study in Swaziland looked at the efficacy of LabPush, an SMS test reporting tool, compared to traditional paper reports (34). SMS delivery of results was at least 1 day faster for 49% of results, although for 28%, paper results arrived first. Furthermore, 99% of SMS test results were received, compared to 92% of paper results, showing a reduction in lost results. Non-automated SMS reporting was, however, identified as a cause of reporting delays in a TB study in Cambodia (56).

Birth registration can ‘enhance health systems, increase accountability, and reduce mortality’ (72), yet approximately a third of births worldwide are not registered (83). Schemes implemented in Uganda, Senegal, and Brazil have shown that community workers equipped with mobile technologies can improve coverage of birth registration to nearly 100% (67). A scheme in Kenya encouraged village elders to record the weight of newborn infants within 7 days of birth. Whilst improved data on birth weights were recorded, importantly the scheme also noted an improved pregnancy case discovery before delivery, with only 25% of pregnancies discovered after delivery. This was a significant reduction from 30% 6 months earlier, with important implications for antenatal care (18).

Only a third of deaths worldwide have a medically certified cause of death (75). Verbal autopsies (VA) aim to assign cause of a death from data collected by interviewing family members. Paper-based VAs can be assessed by doctors to assign causes of death, but this can be slow and inconsistent. Mobile InterVA (MIVA) is a smart phone based VA tool which both guides VA interviews and computes likely causes of death (30). Results from a pilot in South Africa demonstrated that the MIVA performed better than paper-based VAs, reducing interview times, eliminating paper processing and storage, and making cause of death data immediately available. How to handle and utilise such data is currently an issue for more ethical consideration. It was suggested the quicker determination of cause of death may be important in terms of informing outbreak control and other public health actions.

### Electronic health records

Health records are generally problematic in LMICs, and there were only four mHealth applications in this domain meeting our review criteria. Electronic health records contain key patient conditions, results, and treatment plans. Access to this information facilitates more effective treatment. Consequently some LMICs have piloted electronic health records (27). The next challenge is to see if these can be accessed by mobile devices, to facilitate electronic health records in locations without computer access.

In India, the ‘104 mobile’ scheme sent mobile medical units (MMUs) to rural, medically underserved areas in Andhra Pradesh. This scheme, intended to improve health coverage, was also able to establish 10 million unique electronic health records that would not otherwise have existed (4).

A centralised electronic and mobile-based medical record system for HIV in Rwanda since 2004 has been shown to be successful in improving the quality and speed of data collection, even in the most remote clinics (29, 47).

### Electronic decision support: information, protocols, algorithms, checklists

This domain is largely intended to support and improve the functionality of health systems, by embedding information in mHealth applications, and examples were reported in seven papers. A randomised controlled trial in Kenya looked into malarial treatment adherence across 107 rural health facilities (24). Over 6 months, two SMS messages were sent daily (excluding weekends) to health workers, containing messages on key outpatient management of malaria in an effort to improve the quality of treatment delivered. Results suggested that, in the short term, treatment adherence improved by 31.7% (95% CI 15.6–47.8) and, in the long term, treatment adherence improved by 28.6% (95% CI 12.7–44.6).

CHWs are frequently the main providers of primary care in LMICs, but care quality can be poor (12). A randomised prospective crossover simulation study demonstrated that mHealth-based guidelines support reduced error rates by 33% (*p*=0.0001) and improved compliance to protocols by 30% (*p*=0.001) (68). Additional testing demonstrated mHealth decision aids also reduced mental demand, frustration, and overall workload compared to paper aids (69). These studies were, however, based on simulated case studies and not tested on health problems in a realistic setting.

Case detection of communicable diseases is an important strategy for controlling infection rates in LMICs. A year-long Pakistani communications campaign, aimed at improving TB detection rates, advised patients who had a history of cough lasting at least 2 weeks to present to one of 55 private medical facilities. After arrival, patients were screened by community lay-people using a mobile phone-based algorithm to assess both patients and visitors for risk of TB (62). The lay screeners were incentivised with cash rewards for case detection. Results, compared with an adjacent control region, showed a 2.2-fold increase in TB case detection (95% CI 1.9–2.5), showing a substantial increase in case detection rates. This demonstrated the use of both financial incentives, and the benefits of employing lay people equipped with mobile phones to improve case detection of TB.

The Government of Malawi, in collaboration with UNICEF and Columbia University, developed an SMS-based decision support system to improve the community management of child malnutrition (46). Health workers in the community submitted child nutrition indicators to a central server via SMS which were automatically analysed for signs and symptoms of malnutrition. Instant responses containing guidance on management were received if malnutrition was suspected. Results showed that delays of several months in transporting paper-based data were eliminated. Data quality improved with an average error rate of 2.8% compared to 14.2% in previous paper tools.

A two-arm comparative crossover study of physicians in Botswana aimed to establish the efficacy of information retrieved via mobile phones from medical apps, compared to the website ‘PubMed for Handhelds’ (15). Questions were based on eight scenarios, with the primary outcome being a grade for each question. Results suggested that the use of medical apps led to a higher percentage of fully correct responses compared to PubMed (range 33–63% vs. 12–13%: Apps vs. PubMed). The authors suggested that the condensed content of medical apps was ‘more appropriate for point-of-care needs’.

### Provider-provider communication: user groups, consultation

This is similar in some ways to the previous domain, but relies on mHealth applications to facilitate communication between providers rather than making information available within applications. Eight papers exemplified this. The non-governmental organisation, Worldvision, implemented a scheme in Indonesia that gave mobile technology to midwives in the rural Aceh Besar region to improve point-of-care support (58, 59). Focus group discussions and in-depth interviews (*n*=86) showed benefits, including the facilitation of communication, greater time efficiency, and better access to medical information. A quantitative before and after survey showed that, at endline, midwives were more likely to consult specialists and access health information from the health centre using their mobile phones while providing emergency care, than at baseline. Furthermore analysis showed 92% of midwives felt more confident at tackling complex obstetric emergencies (*p*<0.10). Although data on overall improvements in mortality for each scheme were unavailable, improved utilisation of specialist birth attendants was also observed.

A system using SMS and voice support from physicians to CHWs has been used in rural Rwanda (28) in an effort to improve maternal care. CHWs (*n*=432) were given mobile phones and trained to use them in conjunction with antenatal follow up in the community. At the end of the pilot study, results showed that facility-based delivery improved by 27% (compared to 12 months previously), to a coverage of 92% in total.

The Indian governmental Health Management and Research Institute launched a public-private partnership in India called ‘104 mobile’, in 2008, in the state of Andhra Pradesh. It works to support primary care workers in rural areas (81). The scheme aims to provide disease prevention counselling, supply chain management, telemedicine, and disease surveillance via mobile technologies through MMUs (4). MMUs are sent to rural areas more than 3 km from the nearest public health centre. At the point of delivery there are trained paramedics, pharmacists, and doctors; whilst in the support centre there are trained specialists in a variety of fields to offer advice via mobile telephone and mobile Internet pathways to both patients and doctors on the front line (66). The main services offered are antenatal check-ups, height and weight monitoring, basic blood and urine analysis and screening, and medicine dispensary, free of cost for 1 month. In 22 districts of Andhra Pradesh, 475 units were sent out to expand the coverage of the health service by 25% relative to baseline levels. The service grew rapidly from four specialists serving about 200 calls a day to 400 serving about 50,000 phone calls daily. Overall this service achieved impressive results with 1.26 million pregnant women receiving three antenatal care check-ups; 55% of 600,000 people, whose needs would otherwise have been unmet, received outpatient treatment (via phone); and 10 million unique electronic health records being established.

St Gabriel's Hospital in Malawi piloted a scheme to reduce unnecessary trips between rural communities and hospitals, with some communities being situated more than 150 km from the nearest hospital. Seventy-five CHWs, generally volunteers from villages, were given mobile phones and trained to use them in situations pertaining to patient adherence reporting, appointment reminders, and communication with physicians (25). This intervention was focused on ameliorating the barriers of poor doctor-patient ratio and distance to hospitals via CHW mediation. Overall the pilot reported promising results with an overall saving of US$ 2,750, mainly through reduced fuel costs. Though not specifically a TB intervention, it is notable that coverage of TB treatment was effectively doubled because of an increase in time available to CHWs. It is important to note, however, that this evaluation was based on a retrospective observational study so was not placed under the rigors of a randomised controlled trial, with recall bias noted as a major problem. The report does argue however that ‘it is impossible to ignore the numerical increases in the tuberculosis treatment programme, for which there exist long-standing, well-catalogued records’.

A mixed methods cluster-randomised trial sub-study on mHealth support for HIV CHWs in rural Uganda involved the intervention arm of CHWs (*n*=13) being given mobile phones and encouraged to contact a clinic hotline with any concerns they may have had (39). Clinic staff could offer advice via phone, arrange for higher-level medical care to review the patient, or arrange for transport to health facilities. Qualitative results suggest improved communication and patient care, because patients could now contact CHWs who would work to treat the patient (if possible), whereas previously their needs would have gone unmet or be borne by clinic staff. Quantitative results from the 970 patients managed (446 in intervention arm, 524 in control), however, failed to show any statistical difference between the intervention and the control arm in terms of the primary outcome (virological failure) (19.4% vs. 16.4% in the intervention and control arms, respectively).

### Provider work planning and scheduling

This domain covers management support to providers in particular circumstances, of which four examples were found. Malaria prevention and control is vital to reducing the burden of disease in LMICs. The ‘Better Border Healthcare Programme’ on the Thai-Myanmar border provided malaria staff at treatment sites with mobile telephones (64). Each phone was preloaded with software that generated a follow-up schedule after initial treatment was administered to patients diagnosed with malaria. Results showed that in Thai patients, compared to the baseline of 20–40% before the intervention, follow-up rates post-intervention stood at greater than 90%. Results improved to a follow-up rate of more than 80% amongst patients in the immigrant community too, compared to a baseline of less than 10% before the intervention. The authors concluded that mobile technology improved the speed of data collection compared to paper processes, and improved follow-up rates and malaria control actions.

In Sao Paolo, Brazil, automated appointment reminders were sent to patients across four health centres (51). Attendance rates of those sent reminders were compared to the control arm of those who did not receive reminders. Results indicated a significant reduction in the mean non-attendance rates from 25.6 to 19.4%. Nigerian cancer patients (*n*=1,160) enrolled in a scheme whereby they were given the medical team's mobile telephone number to facilitate communication with and from the cancer service, were compared with a control group (*n*=219) who did not have phone access. After 2 years, only 19.2% of the non-mobile phone patients had maintained their appointments, compared to 97.6% in the intervention group (26). More than 25 calls each were made by almost 500 of the patients.

A cross-sectional and prospective study of HIV-positive people in rural Uganda demonstrated that 79% of patients who had missed an appointment, and were reminded via SMS or voice calls, presented for appointments within 2.2 days (43). On the other hand, a voice call appointment reminder scheme study in Swaziland offered no statistically significant improvement to HIV appointment attendance (35).

With a focus on the health service supply side factors that can result in missed appointments, the ‘CommCare’ project in Tanzania utilised SMS messages to remind CHWs about appointments the preceding day and on the day of routine health visits. If an appointment was missed, daily reminders were sent, with an upgrade to a voice call reminder from a supervisor if the appointment remained overdue by 3 days (36). Results from a small experimental pilot (*n*=15) showed, that reminders led to a reduction in the average number of overdue days for visitations to 1.4 days in intervention areas compared to a baseline rate of 9.7 days.

### Provider training and education

This domain is a hybrid between mHealth and mLearning, covering training applications in the health sector, of which there were five examples. Several studies have been undertaken that indicate high levels of satisfaction among users of mLearning tools for medical education (14, 55). Short-term learning outcome analysis has shown the benefits of access to medical apps with condensed medical information in Botswana, but does not analyse the long-term benefits to knowledge retention (15). SMS delivery of medical abstracts in response to SMS queries has been demonstrated to be useful in areas with poor mobile Internet access in Botswana (13). A study of 223 midwives in Indonesia showed mobile technology improves access to medical information resources, which is positively associated with improved health knowledge (59).

### Supply chain management

Supply chains for medications, vaccines, and other materials are a major issue in many LMICs, so tools to assist in this area could be important. We found five examples. UNICEF Uganda reports the development of their ‘mTRAC’ system. Health facility workers send information on medication stocks to the government. mTRAC aims to improve drug stocks, and improve the transparency and accountability for medications (49). Analysis of mTRAC showed a reduction in stock-outs of key malarial drugs from 25 to 14% since its implementation (50).

Counterfeit drugs have become a global problem, not least in LMICs (60). mHealth tools have been developed to track counterfeit and poor quality drugs. A handheld device developed in the United States detects differences in counterfeit packaging and is currently undergoing field tests in Ghana (80). A US based company, Sproxil, has developed a system that aims to improve the authenticity of medications. Customers scratch off a panel on drug packaging, revealing a code which can be sent via SMS to verify authenticity. According to the New York Times, this has led to 9 million verifications being undertaken since the project was launched in 2010 (82).

## Discussion

mHealth is clearly becoming an important concept in LMICs, but as yet there is very limited hard evidence on its effects within health systems. Such evidence as there is largely concerns pilot studies and small-scale implementations, and is not completely positive in terms of potential mHealth benefits. Much of the available evidence is somewhat anecdotal and health service providers in LMICs are, as yet, in a difficult position in terms of choosing effective and cost-effective mHealth strategies for widespread use. Nevertheless, there are many examples which show considerable potential. Experience from Nigeria, where the mHealth intervention was as simple and inexpensive as giving specialists’ contact phone numbers to cancer patients, appeared to achieve considerable benefits (26).

There was considerable variety in the quantity and quality of content across the application domains (8) under which we categorised the 76 publications in the review. Data collection and reporting (including vital event registration) continues to be a major mHealth issue in LMICs. Despite debates that started 25 years ago on the relative merits of paper-based and device-based primary data capture (84), there is still no clear consensus. Because health services in LMICs generally struggle with inadequate data on the populations they seek to serve, this continues to be a major constraint on standardising procedures.

First, client-based interventions for education and behaviour change are a rapidly increasing area of interest, now that substantial proportions of people in many LMIC populations have access to mobile communications technology. Nevertheless, as noted in some of the reviewed papers, attention has to be given to equity issues if mHealth strategies assume the availability of mobile phones, with the attendant risks of further marginalising already vulnerable groups (41, 77). Nevertheless, SMS reminder systems for appointments have improved appointment adherence, which is likely to improve service quality and efficiency of health systems.

The second major grouping is largely technical; applications involving imaging, data collection, registration procedures, and patient records may well benefit from mHealth components, but also need wider functionality in health systems to be beneficial.

The third major area is in mHealth tools that directly support health workers. These range across domains that provide information and decision support to professionals, which may be beneficial in terms of technical and managerial issues, enable more effective communication with clients, and directly enhance logistic issues such as supply chains.

Our review covers the scope of mHealth as postulated by Labrique et al. (8) as comprehensively as possible. The history of mHealth, framed as a potentially important solution to pre-existing problems, so far only covers 5 years. Perhaps this partly explains, despite notable exceptions, why at least in LMICs there remains a strong focus on mHealth pilot studies, which have rarely been followed-up with more rigorous evaluation studies and have generally not been taken to scale. This seems to be a major weakness, which threatens the future credibility of mHealth as a concept. If the impact of mHealth interventions is to be adequately understood, and promising initiatives scaled-up, it is imperative to undertake more rigorous evaluations. mHealth interventions need to be proven to be effective and cost-effective before they can be implemented on a routine basis, as is the case for any other health service technology or intervention. Hopefully the next 5 years of mHealth will bring more substantive developments.
